# The Role of Insulin and Insulin-like Growth Factors in the Increased Risk of Cancer in Diabetes

**DOI:** 10.5041/RMMJ.10043

**Published:** 2011-04-30

**Authors:** Derek LeRoith, Eyal J. Scheinman, Keren Bitton-Worms

**Affiliations:** “Diabetes and Metabolism Clinical Research Center of Excellence”, Legacy Heritage Clinical Research Institute at Rambam (LHCRIR), Haifa, Israel

**Keywords:** insulin, insulin-like growth factor-1, cancer, diabetes, insulin resistance, hyperinsulinemia

## Abstract

Patients with type 2 diabetes (T2D) are at increased risk of developing cancer. This evidence arises from numerous epidemiologic studies that relate a positive association between T2D and cancer. *In-vitro* and several *in-vivo* experiments have attempted to discern the potential mechanistic factors involved in this relationship. Candidates include hyperinsulinemia, insulin-like growth factor-1 (IGF-1), and insulin-like growth factor-2 (IGF-2) signaling. These studies demonstrated that increased insulin, IGF-1, and IGF-2 signaling through the insulin receptor and IGF-1 receptor can induce cancer development and progression.

## INTRODUCTION

Type 2 diabetes (T2D), a growing epidemic seen worldwide, is associated with an increased risk of developing cancer and increased cancer-related mortality. T2D is characterized by insulin resistance, hyperglycemia, hyperlipidemia, and hyperinsulinemia. Studies using diabetic non-obese mice demonstrated the link between diabetes and tumor development and metastasis, which is dependent on insulin and insulin growth factor-1 (IGF-1). In this paper we will discuss the epidemiologic evidence for a link between diabetes and cancer and the potential mechanisms involved. We will focus mainly on insulin and IGF-1 and their receptors, and the molecular mechanisms that are involved in their activation.

## EPIDEMIOLOGY

Diabetes, particularly type 2 diabetes (T2D) is a growing epidemic worldwide. The increase in cases of T2D is apparently driven by the epidemic in obesity. In 2005, diabetes was the third leading cause of death among people between the ages of 45 and 64 in Israel.^1^ It is considered to be one of the leading causes for morbidity and death from cardiovascular disease.^1^ According to the Center for Disease Control and Prevention (CDC), 1 in 10 adults in the U.S. now has diabetes, and by the year 2050 it could rise to between 1 in 3 to 1 in 5 adults, if life-style changes are not adhered to sufficiently.^2^ T2D is a polygenic disorder that is strongly influenced by obesity, which leads to insulin resistance and β-cell dysfunction, and is reflected by hyperinsulinemia and hyperglycemias.^3^ Epidemiological studies have shown that both obesity and T2D are associated with an increased risk of developing various cancers as well as an increase in cancer-related mortality.^4^ T2D was found to increase the risk for one of the most common cancers all over the world, namely breast cancer.^5^–^8^ Hyperinsulinemia was identified as the predominant factor of mammary tumor progression mediated by diabetes.^9^ It was found that, regardless of their body mass index (BMI), more patients with T2D developed cancer and demonstrated cancer-related mortality.^5^,^10^

## DIABETES AND CANCER

Data acquired by epidemiological studies in recent years support the idea that there is an association between T2D, obesity and cancer.^11^ It was found that the relative risk of overweight men with BMI ≥ 35 kg/m^2^ to die from cancer is 1.23 compared with normal-weight men with BMIs between 18.5 and 24.9 kg/m^2^. The same ratio was found in women with BMIs of 30–34.9 kg/m^2^ as compared to normal-weight women, and it was increased to 1.62 with a BMI ≥ 40 kg/m^2^.^4^,^11^ Several reports from all over the world pointed out that a BMI ≥ 30 kg/m^2^ raised the odds for all kinds of cancers to 1.29 for men and 1.41 for women.^11^–^15^

Several in-depth studies found that T2D is associated with increased risk of developing cancer, regardless of obesity.^11^ These studies distinguished the cancer sites related to T2D such as pancreatic cancer,^16^ extrahepatic biliary cancer,^17^ breast cancer, especially estrogen receptor-positive breast cancers;^18^,^19^ but it seems to have no effect on the incidence of premenopausal breast cancer.^19^ No link was found between T2D and lung cancer.^8^,^20^

Some of the studies carried out in order to draw a connection between diabetes and cancer do not distinguish between type 1 and 2 diabetes and describe only the existence of diabetes at the time of diagnosis of the cancer. However, the majority of studies suggest the connection is between T2D and cancer. Several meta-analyses thus attempt to discern the cause for cancer development and to distinguish between the two types of diabetes as well as other factors such as hepatitis for liver cancer and smoking for lung cancer. The overall conclusion from these meta-analyses was that there is sufficient evidence to conclude that an association exists between T2D and the risk for several types of cancer including breast, colorectal, pancreatic, and bladder cancer. However, the opposite was found in the case of prostate cancer.^21^–^27^

Insulin resistance and hyperinsulinemia are associated with obesity, the latter leading to T2D in genetically predisposed individuals. Some epidemiological studies demonstrate a direct correlation between insulin and C-peptide levels and cancer development, especially in obese individuals. In one study, it was found that C-peptide base-line levels were significantly higher in men who developed colorectal cancer in comparison to controls, in the absence of T2D.^28^ Studies conducted by the Women’s Health Initiative (WHI) also found a strong correlation between fasting insulin levels and breast and endometrial cancer. These studies pointed out that in women not taking hormone replacement therapy the correlation was even more significant.^29^,^30^

## INSULIN, INSULIN SECRETAGOGUES, METFORMIN, AND CANCER

Epidemiological evidence collected in several studies has found that higher insulin levels may lead to cancer development. Other studies pointed out that use of insulin or insulin secretagogues might increase the risk to develop cancer in certain individuals.^11^ Studies in Canada showed that use of sulfonylurea and insulin elevated the risk for cancer-related mortality compared to T2D patients that were treated with metformin alone. These results are controversial since it was unclear whether the increased mortality risk is a result of use of both insulin and sulfonylurea, or that metformin use decreased the risk.^31^ Other studies found that not only did the use of sulfonylurea and insulin increase the risk for cancer-related death as compared with metformin use, it also increased the risk for cancer development. When metformin was administrated together with insulin or sulfonylurea this effect was decreased.^32^ The Zwolle Outpatient Diabetes Project Integrating Available Care 16 (ZODIAC-16) study conducted in the Netherlands followed diabetic patients receiving insulin, sulfonylurea, or metformin, for 9 years. Their findings were that treatmerit with metformin decreased cancer mortality by 50% in comparison to the other groups.^33^

## THE INSULIN AND INSULIN-LIKE GROWTH FACTOR (IGF) SYSTEM

The epidemiology studies discussed in the first part of this review show an association between T2D and cancer, but in order to understand the causative factors we will first focus on the potential players and their signaling system.

Insulin and insulin-like growth factor (IGF) are growth factors that are involved in various cellular processes: glucose metabolism, cell proliferation, differentiation, and survival.^34^ The insulin and IGF system is a complex network of ligands, receptors, and signaling pathways.

## INSULIN RECEPTOR SIGNALING

**Figure 1:**
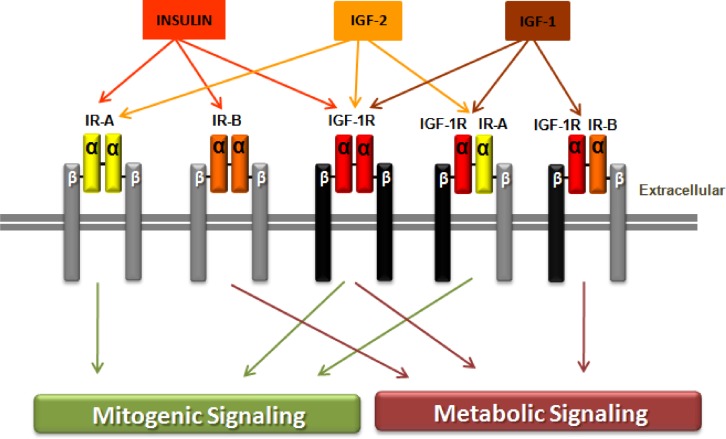
The insulin receptor (IR) with its two subtypes IR-A and IR-B, the insulin growth factor 1 receptor (IGF-1R) and the hybrid receptors (IGF-1R/IR-A and IGF-1R/IR-B). *Structurally,* IR and the IGF-1R have two extracellular α-subunits and two transmembrane β-subunits that are joined to each other by disulfide bonds. *Affinity,* insulin binds with high affinity to IR-A or IR-B but has low affinity for IGF-1R, while insulin has no binding to the hybrid receptors. IGF-1 binds to the IGF-1R and to the hybrid receptors IGF-1R/IR-A or IGF-1R/IR-B. IGF-2 binds to IR-A, IGF-1R, or to IGF-1R/IR-A hybrid receptor. *Signaling,* ligand binding to insulin receptor-A or to IGF-1 receptor mediates the mitogenic signaling pathway, while ligand binding to insulin receptor-B activates metabolic signaling. Binding to the hybrid receptors, leading to mitogenic or metabolic signaling, is determined by the IR isoform that formed the hybrid receptors.

The hormone insulin is secreted mainly by the β-cells from the islets of Langerhans in the pancreas in response to elevation in glucose levels. The main insulin target tissues are liver, skeletal muscle, and adipose tissue. In these tissues insulin has a metabolic effect, whereas high levels of the insulin receptor (IR) in the brain and lower levels in pancreas, monocytes, granulocytes, erythrocytes, endothelial cells, and fibroblasts^35^ suggest that insulin has other roles as well. Insulin binds to the extracellular portion of the transmembrane tyrosine kinase IR. Structurally, IR has two extracellular α-subunits and two transmembrane β-subunits that are joined to each other by disulfide bonds (Figure 1). In addition, alternative splicing yields two IR isoforms: isoform A (IR-A) lacking exon 11, and isoform B (IR-B) including exon 11. Upon insulin binding, autophosphorylation of the β-subunit leads to phosphorylation of intracellular proteins such as insulin receptor substrates (IRS-1 to 4) and other adaptor proteins. Phosphorylation of IRS-1 activates the phosphatidylinositol 3-kinase (PI3K) cascade which turns on the protein kinase B (Akt) pathway. IR activation also activates the mitogen-activated protein kinase (MAPK) pathway.

## IGF-1 RECEPTOR SIGNALING

**Figure 2:**
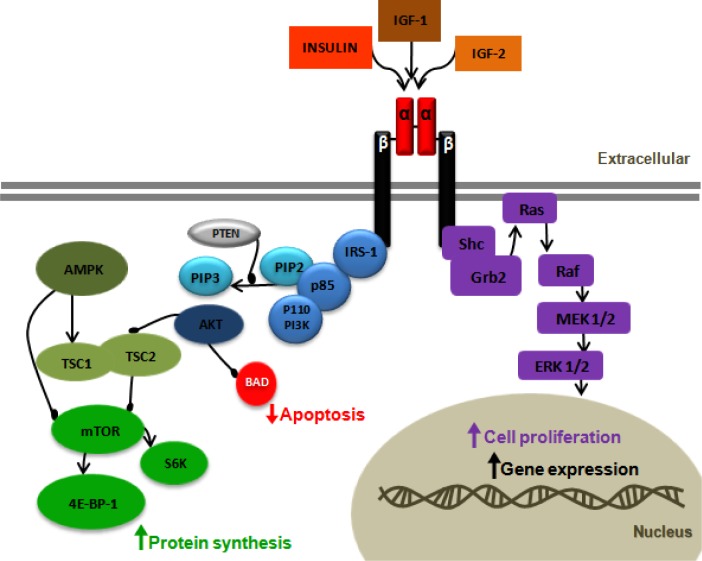
Insulin-like growth factor 1 receptor (IGF-1R) signaling pathway. Binding of IGF-1 or IGF-2 or insulin to the IGF-1R α-subunit leads to autophosphorylation of β-subunit residues, which then act as docking site to insulin receptor substrates (IRS-1 to 4). IRS-1 recruits the p85 regulatory subunit of phosphatidylinositol 3-kinase (PI3K) which activates the p110 catalytic subunit, then resulting in the formation of phosphatidylinositol 3,4 phosphate (PIP2) and phosphatidylinositol 3,4,5 phosphate (PIP3). PIP3 activates Akt. Activated Akt has many substrates; in one pathway Akt inhibits apoptosis by inactivating BCL-2 antagonist of cell death (BAD), and in the second pathway Akt regulates protein synthesis by phosphorylating tuberous sclerosis complex (TSC1/2). This phosphorylation removes the inhibition of TSC1/2 from mammalian target of rapamycin (mTOR). mTOR activates the ribosomal S6 kinase (S6K) and eukaryotic initiation factor 4E-binding protein-1 (4E-BP-1), leading to protein synthesis. In the absence of cellular nutrients, AMPK can inhibit protein synthesis through mTOR inhibition, both directly and by activating the TSC1/2 complex. The tumor suppressor phosphates and tensin homolog deleted on chromosome 10 (PTEN) inhibits PI3K. The mitogen-activated protein kinase (MAPK) pathway can also be activated by IGF-1R activation. In this pathway IGF-1R activates the adaptor proteins, She and Grb2, leading to activation of Ras, Raf, MEK1/2, and ERK1/2, which results in cell proliferation.

Circulating IGF-1 is produced mainly in the liver and responds to growth hormone (GH) stimulation. IGF-1 is also expressed by almost all tissues.^36^ IGFs mainly regulate growth processes and have mitogenic effects. The circulating ligands IGF-1 and IGF-2 are bound to IGF-binding proteins (IGFBPs). There are six IGFBPs, named IGFBP-1 to IGFBP-6; they bind IGF-1 and IGF-2 but not insulin and protect them from degradation. Like insulin, IGF ligands (IGF-1 and IGF-2) bind to a tyrosine kinase receptor, the IGF-1 receptor (IGF-1R). The IGF-1R is similar in structure to the insulin receptor, with two extracellular α-subunits and two transmembrane β-subunits (Figure 1). Binding of IGF-1 or IGF-2 to the IGF-1R leads to receptor autophosphorylation, which results in IRS phosphorylation that then leads to activation of the PI3K cascade. IGF ligand binding can also activate the mitogen-activated protein kinase (MAPK) pathway (Figure 2). In addition, IGF-2 can also bind IGF-2 receptor (IGF-2R) which leads to endocytosis of the ligand–receptor complex, and therefore IGF-2R functions as a clearance receptor for IGF-2.

## INSULIN AND IGF RECEPTORS

Insulin and IGF-1 bind their own receptors at physiological concentrations, but due to their high homology in the structure of their receptors a hybrid receptor may also exist. This may give rise to multiple variations of homo- or heteroreceptor dimers: IR-A/IR-A, IR-B/IR-B, IGF-1R/IGF-1R, IGF-1R/IR-A, and IGF-1R/IR-B (Figure 1). Insulin binds with high affinity to the IR-A or to IR-B but has low affinity for IGF-1R, while insulin has little or no binding to the hybrid receptor.

IGF-1 has high affinity for the IGF-1R and to the hybrid receptors. IGF-2 can bind to IR-A or to IGF-1R and also to the hybrid IGF-1R/IR-A. In addition only IGF-2 can bind to the IGF-2R; this interaction mediates the endocytosis and clearance of IGF-2 from the circulation.^37^ In general, ligand binding to the IR-A or to the IGF-1 receptor mediates the mitogenic signaling pathway (cell survival, growth, and proliferation), while ligand binding to IR-B activates metabolic signaling. Binding to the hybrid receptors, leading to mitogenic or metabolic signaling, is determined by the IR isoform that formed the hybrid receptors (Figure 1).

## ANIMAL MODELS

### IGF-1, IGF-1R, AND CANCER

To understand the relationship between T2D, obesity, and cancer risk, the effects of the insulin and IGF-1 signaling have been studied in animal models of cancer and cancer cell lines. These studies help determine the mechanisms involved.

In mice, IGF-1 levels were reduced by caloric restriction treatment and led to a reduction in tumor growths^8^ In rodents with reduced circulating IGF-1 levels tumor growth and metastasis were reduced. Administration of IGF-1 ligand to these mice reversed the reduction in both tumor growth and metastases.^39^ in addition, in Noble rats (prostate carcinoma model), increased IGF-1 levels resulting from exposure to high levels of sex hormones led to progression from benign prostatic growth to adenocarcinoma of the prostate^40^ IGF-1 signaling appears to prevent apoptosis by up-regulating the expression of MDM2. This protein facilitates P53 inhibition^41^ IGF-1 induces redistribution of integrins, receptors that bind to components of the extracellular matrix and involve cell migration, thereby aiding in metastasis. Addition of IGF-1 to colon cancer cell lines caused re-localization of integrins which resulted in increased cell migration.^42^ Another cell motility feature, the lamellipodia, was found to be induced by IGF-1 in melanoma and neuroblastoma cancer cell lines.^43^ In order to understand the role of the IGF-1R in tumorigenesis, animal studies have investigated modulation of the IGF-1R. Using a transgenic mouse model of adenocarcinoma of the prostate (TRAMP), investigators found high levels of IGF-1 in basal epithelial cells of the prostate that could activate the IGF-1R and result in spontaneous tumorigenesis in prostate epitheliums Other transgenic models that express the IGF-1R constitutively showed aberrant development of the mammary glands and rapid development of salivary and mammary adenocarcinomas.^45^ Expression of an IGF-1R dominant negative mutant in Ewing’s sarcoma cells markedly decreased proliferation and induced apoptosis. When cells expressing a dominant negative IGF-1R were injected into nude mice, the tumor formation and metastatic abilities of the Ewing’s sarcoma cells were reduced, and survival of the mice increased.^46^ In addition, other variations of dominant negative mutations of the IGF-1R in mice blocked the growth of the lung cancer cell line.^47^ IGF-1R over-expressed in a variety of primary cancers increased tumor growth and also increased nodal metastases.^48^ Altogether, these studies suggest that IGF-1 signaling through the IGF-1R plays critical roles in tumor growth, metastasis, and inhibition of pro-apoptotic factors.

## INSULIN RECEPTOR

Similar to the IGF-1R involvement in tumor development, studies involving the insulin receptors indicated a connection between insulin receptors and cancer. Higher levels of IR expression were found in human breast cancer than in normal breast tissue.^49^ Other studies demonstrated that the IR is over-expressed in malignancies such as cancer of the thyroid, colon, lung, ovary, and sarcomas.^50^–^52^ Analysis of five types of human adenocarcinoma (breast, colon, pancreas, lung, and kidney) yielded evidence of higher levels of IR on the endothelium cells. This evidence connects IR over-expression to angiogenesis.^53^ Moreover, *in-vitro* angiogenesis assays that tested various commercially available insulin compounds demonstrated that insulin has the potential to increase capillary-like tube formation of human microvascular endothelial cells (hMVEC).^53^ Similar results were obtained from down-regulation of IR using shRNA. Thus IR inhibition in cancer cell lines (LCC6 and T47D) causes reduced Akt activation by insulin, with no involvement of the IGF-1R. When the cells were transplanted into mice, reduced growth, angiogenesis, and lymphangiogenesis were detected, and reduced expression of hypoxia-inducible factor-1-α(HIF1-α), and vascular endothelia growth factor-A (VEGF-A), and VEGF-D were measured.^54^ Met-1 breast cancer cells that over-express the viral oncogene PyVmT (polyoma virus middle T antigen) show interaction of IGF-1R and IR with the PyVmT that increased with IGF-1 and insulin presence. The interactions enhanced tyrosine phosphorylation of PyVmT and raised recruitment of Src and PLCγ1 to PyVmT. Src and PLCγ1 play a role in tumorigenesis. In this setting Met-1 cells demonstrated increased proliferation, survival, migration, and invasion. Also, Met-1 cells with dysfunction of IR and IGF-1R that were transplanted into the hyperinsulinemic MKR (unique transgenic model of T2D) mice lost the ability to initiate tumor growth.^55^

## IGF-2 AND CANCER

The *igf2* gene is maternally imprinted in mouse and human. *Igf2* gene imprinting is involved in Beckwith–Wiedemann syndrome and Wilms’ tumor.^56^ Transgenic over-expression of IGF-2 in lung epithelium induces lung tumors through IGF-1R signaling pathways.^57^ In a mouse model of colon cancer, IGF-2 increased tumor development on the background of adenomatous polyposis coli (APC) gene mutation.^58^ in addition, IGF-2 can bind to the insulin receptor (IR-A) and activate mitogenic effects.

In summary, all these studies indicate that insulin, IGF-1, IGF-2, and their signaling via the IR and IGF-1R can induce tumor growth.

## INSULIN RESISTANCE AND HYPERIN-SULINEMIA

To decipher the contribution of insulin resistance and hyperinsulinemia in tumor development, we created the MKR mouse model. This model, a dominant negative form of IGF-1R with a point mutation K^1003^➔ R^1003^ is exclusively expressed in the skeletal muscles, resulting in the IR and IGF-1R inactivation. As a result, the receptors failed to stimulate with their ligands, and severe insulin resistance is observed. The female mouse phenotype displays a non-obese phenotype with insulin resistance, hyperinsulinemia, and mild dysglycemia.^59^ When we crossed the MKR model with transgenic PyVmT oncogene (model of mammary tumors), the MKR female mice showed enhanced tumor growth and a more aggressive phenotype of breast cancer compared with control mice. Both tumor tissue and mammary gland demonstrate a higher expression of IR and increased phosphorylation of the IR/IGF-1R and Akt; furthermore, administration of pharmacological blockers of IR and IGF-1R specifically abrogates the accelerated tumor growth.^9^ In conclusion, this study suggested that the IR/IGF-1Rs are the mediators of the tumor-promoting activity of hyperinsulinemia.

## CONCLUSION

The collective evidences from the epidemiological studies and the results of the animal studies demonstrate a link between T2D, obesity, and increased cancer risk and cancer-related mortality. Furthermore, the increased risk is related to increased activation of the insulin and/or IGF-1 receptors and their signaling pathways.

In this paper we focused on hyperinsulinemia and insulin resistance but have not addressed the role of hyperglycemia and hyperlipidemia. Clearly insulin and IGF-1 play major roles in cancer development and progression, especially in obesity and type 2 diabetes. Other potential factors include leptin which is elevated in obesity and has been shown to stimulate cancer cell growth *in vitro.* Adiponectin, a hormone secreted from adipose tissues, and other cytokines, will clearly be targets for further investigations in the case of breast and other common cancers.

## Abbreviations:

BMI: body mass index
CDC: Center for Disease Control and Prevention
HIF1-α: hypoxia-inducible factor-1-α
IR: insulin receptor
IGFBPs: IGF-binding proteins
IGF-1: insulin-like growth factor-1
IGF-1R: IGF-1 receptor
MAPK: mitogen-activated protein kinase
PI3K: phosphatidylinositol 3-kinase
T2D: type 2 diabetes
VEGF: vascular endothelial growth factor
